# The Development of Health Services in Turkey and the Effects of the Pandemic

**DOI:** 10.7759/cureus.77672

**Published:** 2025-01-19

**Authors:** Turan Poyraz

**Affiliations:** 1 Department of Elderly Care, Izmir University of Economics, İzmir, TUR

**Keywords:** brain draine, covid-19 pandemic, global healthcare systems, health economics, health services, social security, turkey

## Abstract

The concept of social security is used to describe the protection mechanisms created at the social level against future partial-risk situations. Turkey's ability to maintain good health outcomes depends on its strong health insurance system. At this point, the new health system will focus more on individual and social protection and will try to keep service use at a more acceptable level. To ensure that the system is sustainable, it is essential to create different coverage packages in line with income and desires within the scope of the Social Security Agency (SSA) and for individuals to act on the path drawn in line with these packages. During the pandemic, the health systems of every country have been given an important test. Many countries with inadequate health infrastructure have not been able to adapt quickly to the pandemic. COVID-19 has highlighted global inequalities, the spread of the epidemic, the adequacy of efforts to combat it, the capacity of health systems, and the insufficiencies in global health cooperation. On the other hand, countries with well-designed health infrastructure were able to respond quickly to the pandemic. Today, healthcare services in Turkey are conducted in public hospitals, private hospitals, private polyclinics, and private physician practices. All healthcare providers continue their services under the supervision and licensing of the Ministry of Health. The expansion of privatization policies in Turkey has brought with it a system based on a consumption economy, dependent on foreign investments. Considering the report data, which also reveal the dimensions of verbal and physical violence that doctors in Turkey have faced throughout their professional lives, the reasons for the inadequacy in the number of doctors per capita in Turkey are revealed more clearly. Therefore, improving the working and living conditions of health workers, especially doctors, is crucial in meeting the increasing demand for doctors in society. With the impact of the COVID-19 pandemic, the aspects of change that have emerged or are likely to occur in the Turkish health system have varied organizationally, sectorally, and socially. While the COVID-19 pandemic has revealed the positive and negative aspects of the individual, communities, and the global system, the Turkish health system has also had the opportunity to make a self-assessment of its current situation. At the macro level, due to the negative impact of COVID-19 on the world economy, Turkey has experienced an increase in the share allocated to health, financial and inflation problems, and difficulty in access to medicines and medical supplies.

## Editorial

The concept of social security is used to describe the protection mechanisms created at the social level against future partial-risk situations [1]. Public institutions manage, operate, and plan these protection mechanisms for future risks. The assurance of this system includes problems and risks such as illness, birth, work accidents, unemployment, incapacity, old age, and death. Social security was conceptually used for the first time, with the "Social Security Act" enacted in the United States of America (USA) in 1935. This law includes social insurance, social assistance, benefits financed by general revenue, and family benefits. The Bismarck government first introduced social health insurance in Germany in 1883. Therefore, health systems in which health services are financed by insurance funds are also called Bismarckian health systems. The first application in Germany was in the form of sickness insurance, and later, in 1884 and 1889, occupational accidents and old-age insurance were added to the system [1]. The insurance system spread to Europe, Latin America, the USA, and Canada in the 1930s and to African and Asian countries after the Second World War. The International Labour Organization (ILO) has played an important role in the development of social security policies.

COVID-19 emerged in Wuhan, Hubei Province, China, in December 2019. In Turkey, the first case was seen on March 11, 2020. On May 5, 2023, it was stated by the World Health Organization that there is no ongoing health problem that does not constitute an emergency in terms of public health at the international level [2]. Immediately after the emergence of the coronavirus, it showed its destructive effect in the economic and social fields around the world and brought social life to a standstill. Undoubtedly, the biggest impact of the pandemic, which disrupted many sectors in terms of both supply and demand, was in the field of health services. The allocation of some of the health institutions to combat the coronavirus and the fact that some of the institutions are working with incomplete capacity has led to a decrease in the supply of health services. In response to the restrictions in the health supply, there is also a decrease in the demand side. Here, the shock effect of the pandemic and the increase in asymmetric information in patients had a negative impact on the demand for health services [3].

Historical development of health services in Turkey: This is divided into two periods: pre- and post-republic. The Seljuk Empire and its successor, the Ottoman Empire, applied advanced health services according to the conditions of their times. In the later periods of the Ottoman Empire, the influence of the monarchy was felt more, and health services were offered to the army with a palace-centered administrative structure. The public, on the other hand, provided health services to self-employed physicians for a fee. The Gülhane Military Hospital, which was opened in 1898 within the territory of Turkey, constituted the first modern hospital that was established in an orderly and regular manner.

The medical education provided here under the leadership of German professors, especially Rieder, is crucial for Turkish medicine [4]. The Ministry of Health was established with a law that entered into force on May 3, 1920, with the opening of the Turkish Grand National Assembly (TGNA). With the establishment of the ministry, the central and provincial organizations were restructured. As the first task, the Ministry of Health aims to determine the priorities of health services, increase the gains in the field of health, and distribute resources according to the determined criteria. During this period, there was no regular registration system regarding health services, focusing on healing war wounds and improving legislative infrastructure. After the Republic was declared, Dr. Refik Saydam became Minister of Health. Saydam, who contributed greatly to the development of health services, gave priority to preventive health services and preferred to guide local governments instead of seeing curative services as the duty of the state. The principles of health policies to be realized have been determined. These include single-handed execution of health services/vertical organization, separation of preventive and curative medicine, the establishment of medical faculties, and fighting against infectious diseases, such as malaria, syphilis, and tuberculosis [4].

Health services are an area that has a great impact on both the economic and social life of a country. Therefore, it is one of the priorities of policymakers and decision-making authorities in all countries.

With the proclamation of the Republic, the new nation-state that emerged from the Ottoman Empire established the Ministry of Health as the first job in Turkey and attempted to heal war wounds. With the establishment of the nation-state, it began to play an active role in health services. In this case, the Keynesian and welfare state policies, which started to develop after the 1929 economic crisis, were also effective [4]. However, during this period, private medicine and even private hospitals always took place. The biggest reason for this situation is that there are different income groups and demands of these groups in the population. Socialization in Health, which emerged under the leadership of Nusret Fişek in 1960, is a project that emerged as a result of the cyclical structure of the period. The changes that started to be expressed during this period, especially with liberal movements from the 1980s, began to take effect with the Health Transformation Program (HTP) in 2003 [4]. The HTP aimed to benefit from the productive structure of the private sector and change the understanding of the right to health as its ultimate goal. The aim was to design a structure in which service provision is private and the state is dominant in terms of insurance. Turkey is currently in a period when a more advanced phase of the HTP is being experienced. The issue of sustainability is now being discussed more intensively, and the methods that can be used to maintain the current outputs are discussed. Turkey's ability to maintain good health outcomes depends on its strong health insurance system. At this point, the new health system will focus more on individual and social protection and will try to keep service use at a more acceptable level. To ensure that the system is sustainable, it is essential to create different coverage packages in line with income and desires within the scope of the Social Security Agency (SSA) and for individuals to act on the path drawn in line with these packages [5].

Today, health services in Turkey are conducted in public hospitals, private hospitals, private polyclinics, and private physician practices. All healthcare providers continue their services under the supervision and licensing of the Ministry of Health. The expansion of privatization policies in Turkey has brought with it a system based on a consumption economy, dependent on foreign investments. On the other hand, primary healthcare services continue to be carried out through family medicine, community health centers, and family health centers, while secondary healthcare services continue to be carried out through state hospitals with a public understanding [5]. However, the fees paid by patients who benefit from general health insurance, under the name of contribution, are increasing daily, excluding general data and premium payments. In addition, a significant portion of the general health insurance budget is provided by the financing transferred to the social security institution through the general tax system. This is the wage that citizens pay indirectly.

During the pandemic process, the health systems of the countries have been given an important test. Many countries with inadequate health infrastructure have not been able to adapt quickly to the pandemic. COVID-19 has highlighted global inequalities, the spread of the epidemic, the adequacy of efforts to combat it, the capacity of health systems, and the insufficiencies in global health cooperation. On the other hand, countries with well-designed health infrastructure were able to respond quickly to the pandemic. With the impact of the COVID-19 pandemic, the aspects of change that have emerged or are likely to occur in the Turkish health system have varied organizationally, sectorally, and socially [6].

The COVID-19 pandemic can be considered a turning point in areas such as business, social life, education, and health. The finding that violent incidents increased and became more frequent during the pandemic period compared to the pre-pandemic period indicates that there is a discrepancy in the patient-healthcare worker relationship [7]. In addition, with this epidemic, hospital managers have gained experience in crisis management. Health managers have a tendency to experience personal fatigue, uncertainty, and stress. Behaviors of trying to overcome institutional challenges such as creating solutions for the shortage of materials, personnel, and services were examined. While the COVID-19 pandemic has revealed the positive and negative aspects of the individual, communities, and the global system, the Turkish health system has also had the opportunity to make a self-assessment of its current situation. At the macro level, due to the negative impact of COVID-19 on the world economy, Turkey has experienced an increase in the share allocated to health, financial and inflation problems, and difficulty in access to medicines and medical supplies.

Worldwide, doctors are defined as professional groups that provide practical health care directly to patients. In some countries, including Turkey, the lack of comparative data between health professionals such as researchers, managers, educators, and doctors working in the health sector causes different variables to play a role in the calculation of the number of doctors per capita. The current data published by the Organization for Economic Co-operation and Development (OECD) provide us with important information about people's access to health services today when the world is experiencing an important health crisis, such as the COVID-19 pandemic [8].

The fact that the increased brain drain in Turkey, especially with the economic crisis, covered a significant part of health workers and doctors, resulted in a decrease in the number of qualified health workers and doctors in Turkey and a decrease in the number of doctors per capita in population.

The “Health at a Glance” data published by the OECD in 2022 and updated on its page during the period makes it possible to see the number of doctors per capita in OECD countries, including Turkey, comparatively [8].

**Figure 1:**
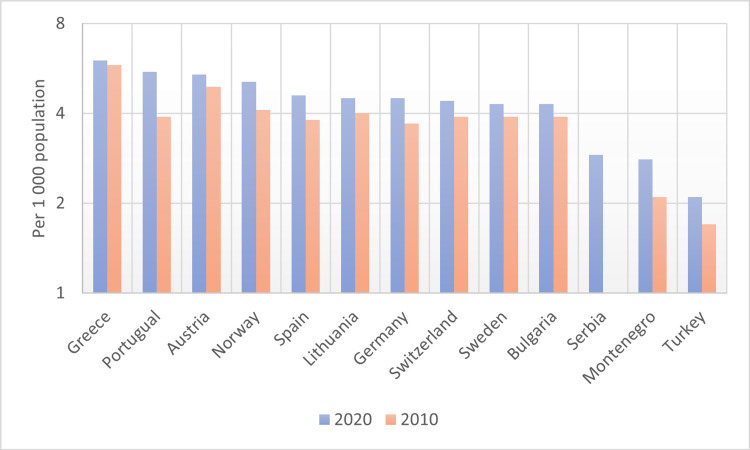
Practising doctors per 1,000 population (2010 and 2020 (or nearest year)) According to the OECD data, the change in the number of physicians per 1,000 people between 2010 and 2020 was shown. The graph and data are created based on this information (figure adapted from Ref. [8]).

According to the OECD data on the number of doctors per 1,000 people in countries, Turkey ranks last among OECD countries with a ratio of two doctors per 1,000 people. In 2020, Greece had the highest number of doctors (6.2 per 1,000 population), followed by Portugal (4.5 per 1,000 population), but the number in these two countries is an over-estimation, as it includes all doctors licensed to practice (including retired physicians and those who might have emigrated to other countries but have kept their license in the country). Therefore, among OECD countries with the highest access to physicians and the lowest number of patients for whom physicians are responsible, Austria ranks first with 5.4, followed by Norway with 5.1, Spain with 4.6, and Germany and Lithuania with 4.5 (Figure 1).

**Figure 2:**
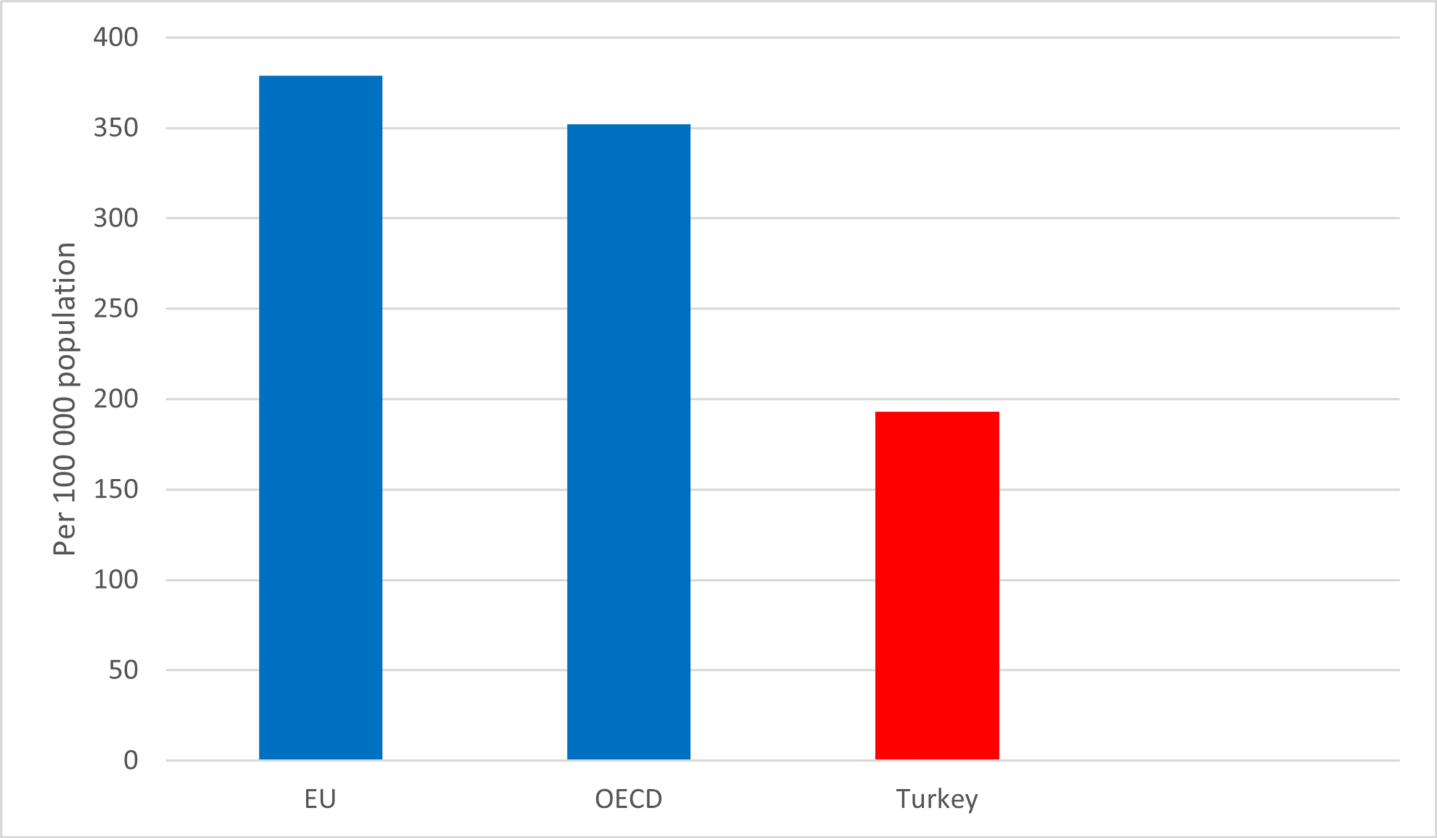
International comparison of the total number of physicians per 100,000 population It has been prepared according to the data in the report titled "Health at a Glance," published by the OECD in 2022, covering the European Union and OECD countries, and the study report data of the Turkish Medical Association, covering the period between 2020 and 2022 (figure adapted from Refs. 5 and 8). EU, European Union; OECD, Organization for Economic Co-operation and Development

Although the number of doctors per capita in Turkey is in last place in the OECD ranking, it is observed that the rate of applying to a doctor in the population has increased over the years. In fact, in the last 20 years, it has been determined that there has been an increase in physician applications per capita of approximately seven times the increase in the number of physicians in the country (345%). Turkey ranks second in terms of annual applications per doctor [5,8]. Turkey remains below the European Union and OECD countries in the International Comparison of the Total Number of Physicians per 100,000 persons (Figure 2).

**Figure 3:**
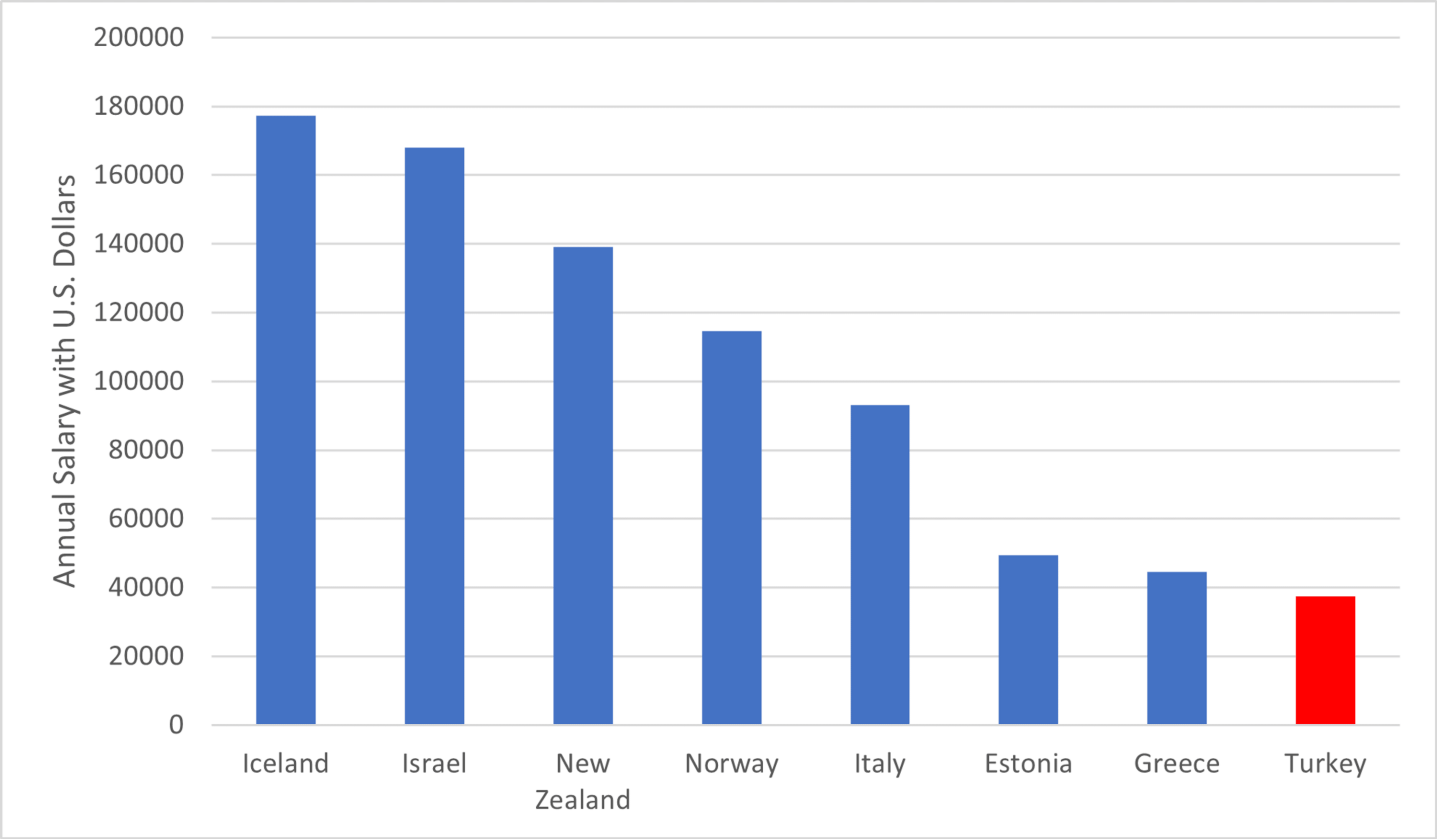
Annual salary of a specialist in various countries It has been prepared according to the data in the report titled "Health at a Glance," published by the OECD in 2022, covering the European Union and OECD countries (figure adapted from Ref. [8]). US: United States

The data revealed that, with the decrease in the number of doctors per capita in Turkey, patients continue to have problems accessing qualified healthcare and health services. The fact that the increase in demand for access to doctors in the population cannot be met by the number of doctors in the country can be identified as a serious problem for our country in the context of the ongoing COVID-19 epidemic crisis. Among the reasons for the low number of doctors per capita in Turkey, it is possible to list the increasing violence against health workers in recent days, the low remuneration of doctors' salaries, and the brain drain factors that cause doctors to reject precarious and low-paid working conditions and prefer to work abroad [5]. The data revealed by the OECD in recent years also supports the effects of these factors. According to OECD data, when the salaries of specialist physicians are analyzed in US dollars, Turkey is ranked 23rd among the 28 countries (Figure 3). The report titled "Quality of Life of Doctors in Turkey,” published by the Turkey Report last year, using data from the Turkish Medical Association, also revealed that the number of doctors who went abroad to work in Turkey reached 4,000 [5].

Considering the report data, which also reveals the dimensions of verbal and physical violence that doctors in Turkey have faced throughout their professional lives, the reasons for the inadequacy in the number of doctors per capita in Turkey are revealed more clearly. Therefore, improving the working and living conditions of health workers, especially doctors, is considered important in meeting the increasing demand for doctors in the population.

All physicians who have completed medical school or their specialty training are expected to complete their duty period, which is called compulsory service, within the periods determined for various regions of the country. An increasing number of Turkish physicians want to work abroad due to reasons such as working hours that are much longer than the specified periods, medical-legal problems with unclear boundaries, the number of patients per physician being much higher than in other European countries, politicized health administrative staff, mobbing, and low-income levels. Despite these difficult conditions, Turkish physicians are making intense efforts and self-sacrificing to maintain health services in the best way possible.

## References

[1] Belek I (2020). [What kind of health system? III not insurance, general tax]. Toplum ve Hekim.

[2] (2025). Statement on the fifteenth meeting of the IHR (2005) Emergency Committee on the COVID-19 pandemic. https://www.who.int/news/item/05-05-2023-statement-on-the-fifteenth-meeting-of-the-international-health-regulations-(2005)-emergency-committee-regarding-the-coronavirus-disease-(covid-19)-pandemic.

[3] Karaca Z, İnan K (2022). [The effect of the pandemic on health services demand: an application on Atatürk University Faculty of Dentistry]. Trends in Business and Economics.

[4] Cavmak S, Cavmak D (2017). [Historical development of health services in Turkey and health transformation program]. Sağlık Yönetimi Dergisi.

[5] Türk Tabipleri Birliği Merkez Konseyi Çalışma Raporu 2020-2022 (2025). [Turkish Medical Association central council working report 2020-2022]. https://www.ttb.org.tr/yayin_goster.php?Guid=79ad5386-ee09-11ec-af02-d9f029f86304.

[6] Erdem R, Alkan A, Çam S (2023). [Post-pandemic change trends in the Turkish health system]. Abant Sosyal Bilimler Dergisi.

[7] Akca N, Kaya M, Sönmez S (2022). [The effect of the pandemic period on violence against health workers: a study on printed media]. Dicle Üniv İktis İdari Bilim Fak Derg.

[8] OECD/European Union (2022). Health at a Glance: Europe 2022: State of Health in the EU Cycle.

